# Risk factors of sleep-disordered breathing and poor asthma control in children with asthma

**DOI:** 10.1186/s12887-024-04762-7

**Published:** 2024-04-30

**Authors:** Minghui Tao, Yanping Zhang, Ling Ding, Donghong Peng

**Affiliations:** 1 Chongqing University FuLing Hospital, No.2 Gaosuntang Road, Fuling District, Chongqing, 408000 P.R. China; 2 The Children’s Hospital of Chongqing Medical University, 136 Zhongshan Second Road, Yuzhong District, Chongqing, 400014 China

**Keywords:** Asthma, Sleep-disordered breathing, Risk factors, Children

## Abstract

**Background:**

Sleep-disordered breathing (SDB) may lead to poor asthma control in children.

**Objective:**

To identify risk factors of SDB in children with asthma and assess its impact on asthma control.

**Methods:**

In this cross-sectional study, we collected data of outpatients with asthma at the Children’s Hospital of Chongqing Medical University from June 2020 to August 2021. The Pediatric Sleep Questionnaire—Sleep-Related Breathing Disorder and the age-appropriate asthma control tests Childhood Asthma Control Test and Test for Respiratory and Asthma Control in Kids were completed.

**Results:**

We enrolled 397 children with a male-to-female ratio of 1.7:1 and a mean age of 5.70 ± 2.53 years. The prevalence of SDB was 21.6%. Allergic rhinitis (odds ratio OR = 3.316), chronic tonsillitis (OR = 2.246), gastroesophageal reflux (OR = 7.518), adenoid hypertrophy (OR = 3.479), recurrent respiratory infections (OR = 2.195), and a family history of snoring (OR = 2.048) were risk factors for the development of combined SDB in children with asthma (*p* < 0.05). Asthma was poorly controlled in 19.6% of the children. SDB (OR = 2.391) and irregular medication use (OR = 2.571) were risk factors for poor asthma control (*p* < 0.05).

**Conclusions:**

Allergic rhinitis, chronic tonsillitis, gastroesophageal reflux, adenoid hypertrophy, recurrent respiratory infections, and a family history of snoring were independent risk factors for the development of SDB in children with asthma. SDB and irregular medication use were independent risk factors for poor asthma control.

## Introduction

Asthma is a common lower airway respiratory disease, characterized by chronic airway inflammation and hyperresponsiveness. The prevalence of asthma has increased by over 50% every decade in China [1–3]. Approximately 4.2% of the population over 4 years of age suffers from asthma. Despite evidence-based assessment and treatment guidelines, asthma is not well-controlled in over 20% of children [4], posing an enormous economic and mental health burden on children and their caregivers. Further, poorly controlled asthma is a leading cause of emergency treatment, hospitalization, and death of children.

Sleep disordered breathing ( SDB ) is a series of diseases that interfere with normal ventilation and sleep structure, including central sleep apnea syndrome, obstructive sleep apnea syndrome (OSAS), sleep-related hypopnea disorder, etc. Its clinical manifestations are snoring, apnea, mouth breathing, sleep insecurity, daytime sleepiness, and inattentive concentration. The incidence of primary snoring was 1.5–27.6% [5]. The incidence of OSAS was 1.2–5.7% [6]. The most common cause of SDB in children is adenoid tonsillar hypertrophy, which can be treated by adenotonsillectomy [7].

SDB and asthma are upper and lower airway inflammatory diseases, respectively. According to the united airway disease hypothesis [8], upper and lower airway inflammations often co-exist, and upper airway disease may influence the severity and clinical control of lower airway disease. Thus, SDB is a risk factor for asthma [9–12]. In addition, adenotonsillectomy can improve asthma symptoms [13–15]. Although risk factors of SDB in children with asthma have been explored, studies are warranted on combined SDB and other risk factors in children with poorly controlled asthma to help achieve asthma control.

The aim of this study was to determine the correlation between SDB and asthma control level and asthma severity, and analyze the risk factors of asthma combined with SDB in order to identify co affected children earlier and provide more clinical treatment strategies for poorly controlled asthma.

## Materials and methods

### Study subjects

Children aged 0–18 years with asthma were enrolled from June 2020 to August 2021 from the asthma outpatient clinic at the Children’s Hospital of Chongqing Medical University, which is the third-largest children’s hospital in China and mainly serves children in southwest China. Inclusion criteria were: (1) a diagnosis of asthma based on the criteria from the Global Initiative for Asthma (GINA), 2020; and (2) a follow-up duration > 1 month. Exclusion criteria were: (1) a history of chronic lung disease, such as bronchiectasis; (2) a history of adenoidectomy or tonsillectomy; (3) presence of other chronic underlying diseases (e.g., heart disease; chronic lung disease in preterm infants; cerebral palsy; neuromuscular disease; genetic metabolic diseases, such as diabetes mellitus; glycogen accumulation disease; chromosomal disorders, such as trisomy 21 syndrome); (4) presence of craniofacial anomalies; and (5) incomplete questionnaires.

### Data collection

The patients or their guardians completed the Pediatric Sleep Questionnaire—Sleep-Related Breathing Disorder (PSQ-SRBD). The patients underwent an age-appropriate asthma control test and the pulmonary function test if they were able to perform. The patients’ demographic characteristics, medical history (including concomitant atopic diseases), physical findings, PSQ-SRBD findings, asthma control test results, and allergic history were collected.

### Research methodology

#### Anthropometric indicators

Trained technicians measured the patients’ height and weight using standardized protocols and calibrated equipment. According to the standardized growth values and curves for the body weight for length and body mass index (BMI) of Chinese children < 7 years of age [16], “obese” was defined as a body weight for length > P97 in children < 2 years of age and BMI > P95 in children ≥ 2 years of age.

#### Spirometry

The pulmonary function test was performed for children who could undergo it, using the MasterScreen Paed spirometer (Jaeger, Germany). The test’s operation, quality control, and determination of results (nature, type, and severity of disease) followed the Guidelines for Children’s Pulmonary Function Series (II): Lung Volume and Ventilatory Function [17]. The children stood upright, and a nasal clip was used. They completed the respiratory maneuvers under the technician’s guidance 3–8 times. The best performance value was regarded as the test result.

#### Skin prick test

An allergen skin prick solution (Beijing Xinhua Macro-Union Pharmaceutical Ltd. Corp.) was used to detect inhaled allergens, including house dust mites, dust mites, cat feathers, dog feathers, cotton wool, cockroach, birch pollen, corn pollen, cigarette smoke, *Artemisia vulgaris*, yeasts, penicillium, and edible allergens, including peanut, milk, egg, mango, apple, soybean, scallop, beef, shrimp, and crab. Histamine and saline were used as positive and negative controls, respectively. A wheal diameter ≥ 3 mm was considered as a positive reaction [18].

#### PSQ-SRBD

The PSQ is a screening questionnaire recommended by the American Academy of Pediatrics in the 2012 OSAS guidelines [6]. It is a valid, reliable, convenient, and feasible questionnaire covering sleep snoring, sleepiness, and hyperactivity [7, 19]. A PSQ-SRBD score > 0.33 suggests a high risk of SDB. The study subjects were divided into SDB-positive (score > 0.33) and SDB-negative (score ≤ 0.33) groups.

#### Asthma control in children

The Childhood Asthma Control Test (c-ACT) is a questionnaire recommended for children ≥ 4 years of age by GINA to assess asthma control in the past 4 weeks [20, 21]. The Test for Respiratory and Asthma Control in Kids (TRACK) is an assessment tool for asthma control in children < 5 years of age that was developed by American scholars in 2007 and modified for international use [22].

In this study, the official Chinese versions of TRACK and c-ACT were used for children < 4 and ≥ 4 years of age, respectively. The children and their guardians answered the questions in their respective sections separately, and the sum of scores was used for analyses. Well- and poorly controlled asthma groups were defined as a TRACK score ≥ 80 or c-ACT score ≥ 20 and a TRACK score < 80 or c-ACT score < 20, respectively.

#### Irregular medication use

In this study, Irregular medication use refers to poor medication adherence and incorrect inhalation methods, such as a lack of a suitable fog storage tank, incorrect inhalation speed and force, and air leakage during inhalation of medication.

#### Asthma severity in children

In this study, the severity of asthma was divided into mild intermittent, mild persistent, moderate persistent, and severe persistent according to The Routine for Prevention and Treatment of Bronchial Asthma in Children (for trial) [23].

### Statistical analysis

Statistical analyses were performed using SPSS 21.0. Numeral data are expressed as number (%), with the differences between groups compared using the χ^2^ or Fisher’s exact test. Normally distributed data were compared using the independent-samples *t*-test, while non-normally distributed data were compared using the nonparametric test. The multiple logistic regression analysis was performed to predict risk factors for asthma with SDB and poor asthma control. The strength of association is expressed as the odds ratio (OR) and 95% confidence interval (CI). A *p*-value < 0.05 was considered to be statistically significant.

## Results

### Study population

Of a total of 486 children, 89 were excluded according to the exclusion criteria. Finally, a total of 397 children were enrolled, including 252 (63.5%) boys and 145 (36.5%) girls, with a male-to-female ratio of 1.74:1, and an average age of 5.70 ± 2.53 years. Table 1 shows the demographic and clinical characteristics of the patients.

**Table 1:** Clinical characteristics of children with asthma (*n* = 397)

Variables	
Demographics	
Age (years)	5.70 ± 2.53
Sex, male/female	252 (63.5)/145 (36.5)
Han Chinese ethnicity	365 (91.9)
Urban residence	326 (82.2)
Cesarean-section birth	222 (55.9)
Premature birth	37 (9.3)
Breastfeeding < 6 months	124 (31.2)
BMI (kg/m^2^), mean ± SD	16.63 ± 2.39
Allergen test results	
Positive reaction to allergens	237 (59.6)
Allergic skin diseases	197 (49.6)
Comorbidities	
Obesity	62 (15.6)
Allergic rhinitis	214 (53.9)
Chronic tonsillitis	55 (13.8)
Gastroesophageal reflux	11 (2.7)
Respiratory conditions	
Positive PSQ-SRBD findings	86 (21.6)
Adenoid hypertrophy	58 (14.6)
Tonsil enlargement	34 (8.5)
Recurrent respiratory infections	119 (29.9)
Asthma control	
Asthma control	319 (80.3)
Uncontrolled asthma	78 (19.6)
Lung function	
FEV_1_, mean ± SD	98.63% ± 2.93%
FEV_1_/FVC, median (IQR)	90.4% (85.07–94.84%)
Environment	
Maternal smoking	5 (1.2)
Passive-smoking	172 (43.3)
Pet animals	24 (6.0)
Sleep	
Difficulty falling asleep	87 (21.9)
Waking up at night	75 (18.8)
Restless sleep	174 (43.8)
Family history	
Allergic history	133 (33.5)
Snoring history	244 (61.4)
Irregular medication use	107 (26.9)

BMI, body mass index; PSQ-SRBD, Pediatric Sleep Questionnaire—Sleep-Related Breathing Disorder; FEV_1_, forced expiratory volume in 1 s; SD, standard deviation; FVC, forced vital capacity; IQR, interquartile range

Data are expressed as n (%), unless otherwise indicated

### Clinical features of SDB

According to PSQ-SRBD findings, 86 (21.6%) children had a high risk of SDB. Table 2 shows the demographic and clinical characteristics of children with and without a high risk of SDB. The two groups differed significantly in prevalence of allergic rhinitis, chronic tonsillitis, gastroesophageal reflux (GER), allergic skin disease, allergy, adenoid hypertrophy, tonsil hypertrophy, and family snoring history (*p* < 0.05) but did not differ significantly in age, sex, nationality, residence, birth history, breastfeeding duration, or BMI (Table 2).

**Table 2:** Univariate analysis findings of risk factors in children with asthma with or without sleep-disordered breathing (*n* = 397)

Variables	SDB (+) *n* = 86		SDB (-) *n* = 311	*p*-value
Demographics				
Age (years)	6.12 ± 2.70		5.58 ± 2.47	0.077
Sex, male/female	60 (69.8)/20(30.2)		192 (61.7)/119(38.3)	0.171
Han Chinese ethnicity	78 (90.7)		287 (92.3)	
Urban residence	70 (81.4)		256 (82.3)	0.844
Father’s education level				
Low	17 (19.4)		60 (19.3)	0.457
Medium	24 (27.9)		68 (21.9)	
High	45 (52.3)		183 (58.8)	
Mother’s education level				
Low	20 (23.3)		67 (21.5)	0.944
Medium	19 (19.3)		70 (22.5)	
High	47 (54.7)		174 (55.9)	
Cesarean-section birth	56 (65.1)		166 (74.8)	0.052
Premature birth	8 (10.4)		29 (10.7)	0.945
Breastfeeding < 6 months	26 (30.2)		98 (31.5)	0.821
BMI (kg/m^2^), mean ± SD	16.71 ± 2.55		16.61 ± 2.35	0.741
Allergen test results				
Positive reaction to allergens	58 (67.4)		179 (57.6)	0.006
Allergic skin diseases	53 (61.6)		144 (46.3)	0.012
Comorbidities				
Obesity	11 (12.9)		51 (16.4)	0.437
Allergic rhinitis	69 (80.2)		145 (46.6)	< 0.001
Chronic tonsillitis	21 (24.4)		34 (10.9)	0.001
Gastroesophageal reflux	7 (8.1)		4 (1.3)	0.003
Respiratory conditions				
Adenoid hypertrophy	30 (34.9)		28 (9.0)	< 0.001
Tonsil enlargement	17 (19.8)		17 (5.5)	< 0.001
Recurrent respiratory infections	34 (39.5)		85 (27.3)	0.029
Lung function				
FEV_1,_ mean ± SD	98.40 ± 11.90		98.70 ± 12.25	0.861
FEV_1_/FVC, median (IQR)	89.4 (83.93–93.25)		90.56 (85.55–95.19)	0.071
Environment				
Maternal smoking	1 (1.2)		4 (1.3)	1.000
Passive-smoking	38 (44.2)		134 (43.1)	0.856
Pet animals	6 (7.0)		18 (5.8)	0.682
Family history				
Allergic history	29 (33.7)		104 (22.4)	0.961
Snoring history	64 (74.4)		180 (57.9)	0.005

SDB, sleep-disordered breathing; BMI, body mass index; FEV_1_, forced expiratory volume in 1 s; SD, standard deviation; FVC, forced vital capacity; IQR, interquartile range

Data are expressed as n (%), unless otherwise indicated

The multifactorial binary logistic regression analysis revealed that allergic rhinitis (OR = 3.316, 95% CI: 1.721–6.389), GER (OR = 7.518, 95% CI: 1.905–29.664), chronic tonsillitis (OR = 2.246, 95% CI: 1.075–4.693), adenoid hypertrophy (OR = 3.479, 95% CI: 1.708–7.085), recurrent respiratory infections (OR = 2.195, 95% CI: 1.207–3.988), and family snoring history (OR = 2.048, 95% CI: 1.097–3.825) were risk factors for asthma in children with SDB (*p* < 0.05; Table 3).

**Table 3:** Multifactor logistic regression analysis results for children with asthma with sleep-disordered breathing

Variables	OR	95% CI	*p*-value
Age	1.084	0.958–1.225	0.201
Sex	1.579	0.867–2.876	0.135
BMI	1.016	0.899–1.148	0.802
Allergy	0.737	0.35–1.517	0.407
Allergic skin diseases	1.299	0.726–2.324	0.377
Allergic rhinitis	3.316	1.721–6.389	< 0.001
Gastroesophageal reflux	7.518	1.905–29.664	0.004
Chronic tonsillitis	2.246	1.075–4.693	0.031
Adenoid hypertrophy	3.479	1.708–7.085	0.001
Tonsil enlargement	1.710	0.700–4.178	0.239
Recurrent respiratory infections	2.195	1.207–3.988	0.010
Family snoring history	2.048	1.097–3.825	0.024

OR, odds ratio; CI, confidence interval; BMI, body mass index

### Asthma control

According to c-ACT and TRACK results, asthma was poorly controlled in 78 (19.6%) children. There were 107 (26.9%) children did not take medicine regularly for asthma control, in which 65 (60.7%) children missed doses. In the univariate analysis, the well- and poorly controlled asthma groups did not differ significantly in terms of sex, BMI, obesity, allergic rhinitis, chronic tonsillitis, allergic skin disease, allergy, or passive-smoking status (Table 4) but differed significantly in terms of SDB and irregular medication use. Moreover, the prevalence of SDB was higher in the poorly controlled asthma control than in the well-controlled asthma group (34.6% vs. 18.5%; *p* < 0.05).

**Table 4:** Univariate analysis results for risk factors for poorly controlled asthma

Variables	Well-controlled asthma (*n* = 319)	Poorly controlled asthma (*n* = 78)	*p*-value
Demographics			
Age (years)	5.77 ± 2.52	5.40 ± 2.57	0.253
Sex, male/female	198 (62.1)	54 (69.2)	0.239
Han Chinese ethnicity	296(92.8)	69 (88.5)	0.208
Urban residence	260 (881.5)	66 (84.6)	0.520
Father’s education level			
Low	65 (20.4)	12 (15.4)	0.390
Medium	70 (21.9)	22 (28.2)	
High	184 (57.7)	44 (56.4)	
Mother’s education level			
Low	74 (23.2)	13 (16.7)	0.110
Medium	65 (20.4)	24 (30.8)	
High	180 (56.4)	41 (52.6)	
Cesarean-section birth	180 (56.4)	42 (53.8)	0.681
Premature birth	32 (11.5)	5 (7)	0.275
Breastfeeding < 6 months	99 (31.0)	25 (32.1)	0.862
BMI (kg/m^2^), mean ± SD	16.70 ± 2.48	16.36 ± 1.94	0.269
Allergic skin test			
Positive reaction to allergens	194 (60.8)	43 (59.7)	0.280
Allergic skin diseases	158 (49.5)	39 (50)	0.941
Comorbidities			
Obesity	52 (16.4)	10 (12.8)	0.442
Allergic rhinitis	167 (52.4)	47 (60.3)	0.209
Chronic tonsillitis	43 (13.5)	12 (15.4)	0.662
Gastroesophageal reflux	9 (2.8)	2 (2.6)	1.000
Respiratory conditions			
SDB	59 (18.5)	27 (34.6)	0.002
Adenoid hypertrophy	44 (13.8)	14 (17.9)	0.352
Tonsil enlargement	27 (8.5)	7 (9.0)	0.885
Recurrent respiratory infections	97 (30.4)	22 (28.2)	0.704
Environment			
Maternal smoking	4 (1.3)	1 (1.3)	1.000
Passive-smoking	137 (42.9)	35 (44.9)	0.758
Raising animals	21 (6.6)	3 (3.8)	0.595
Sleep			
Difficulty falling asleep	70 (21.9)	17 (21.8)	0.977
Waking up at night	55 (17.2)	20 (25.6)	0.089
Restless sleep	139 (43.6)	35 (44.9)	0.836
Family history			
Allergic history	111 (34.8)	22 (28.2)	0.269
Snoring history	194 (60.8)	50 (64.1)	0.593
Irregular medication use	73 (22.9)	34 (43.6)	< 0.001

BMI, body mass index; SD, standard deviation; SDB, sleep-disordered breathing

Data are expressed as n (%), unless otherwise indicated

In the binary logistics regression analysis, after correcting for mixed factors, such as age, sex, BMI, allergic rhinitis, and allergy, SDB (OR = 2.391, 95% CI: 1.302–4.393) and irregular medication use (OR = 2.571, 95% CI: 1.498–4.414) were independent risk factors for poor asthma control (Table 5).

**Table 5:** Multifactor logistic regression analysis results for poorly controlled asthma

Variables	OR	95% CI	*p*-value
Age	0.924	0.821–1.041	0.193
Sex	1.203	0.690–2.097	0.514
BMI	0.963	0.855–10.85	0.532
Allergy	1.147	0.560–2.349	0.708
SDB	2.391	1.302–4.393	0.005
Irregular medication use	2.571	1.498–4.414	0.001

OR, odds ratio; CI, confidence interval; BMI, body mass index; SDB, sleep-disordered breathing

### Asthma severity

The rank sum test showed that there was a difference in the severity of asthma between the SDB positive group and the SDB negative group ( *p* < 0.05 ). Children in the SDB positive group have more severe asthma(Table 6).

**Table 6:** Correlation analysis between the severity of asthma and SDB

Variables	The average of rank	*p*-value
SDB(-)	190.70	0.014
SDB(+)	219.49	

SDB, sleep-disordered breathing

## Discussion

In this study, asthma was more common in boys, with a male-to-female ratio of 1.74:1, slightly higher than that of 1.5:1 reported in the third epidemiological report on childhood asthma in mainland China [3]. Asthma is often associated with one or more comorbidities. In this study, allergic rhinitis was the most common complication, with a prevalence of approximately 53.6%, consistent with previous reports [24, 25]. Allergic reaction is the main pathogenic factor of asthma. In this study, the prevalence of allergy in children with asthma was 59.6%.

In this study, the prevalence of a high risk of SDB in children with asthma was consistent with previous studies [10, 26, 27], significantly higher than that of SDB in children without asthma (12%) reported in a multicenter epidemiological study in China [28]. This result suggested that children with asthma are more prone to SDB. Asthma is a risk factor for SDB [26, 29–31], probably because of altered sleep architecture and increased respiratory muscle effort in children with asthma, which may aggravate upper airway collapse. Further, the systemic inflammatory response plays a role. Asthma may promote the release of various inflammatory factors, promoting the proliferation of lymphoid tissues in the upper airways, thus aggravating upper airway obstruction. In addition, long-term use of glucocorticoids in children with asthma may contribute to redistribution of fat around the pharynx and reduced local muscle contractile properties, leading to upper airway collapse, thus triggering and aggravating SDB and even OSAS [32, 33].

In this study, the univariate and multivariate analysis results of the PSQ-SRBD findings of SDB-positive and SDB-negative children with asthma revealed that allergic rhinitis, GER, chronic tonsillitis, adenoid hypertrophy, recurrent respiratory infections, and a family history of snoring were independent risk factors for SDB in children with asthma. Compared to children without allergic rhinitis, the risk of SDB was higher in those with allergic rhinitis. This result indicated that allergic rhinitis was a risk factor for SDB in children with asthma, consistent with a previous report [34]. The reason may be that the nasal cavity is the main breathing route during sleep. As the primary clinical manifestation of allergic rhinitis, nasal obstruction increases airway resistance and may lead to mouth breathing, sleep fragmentation, and excessive fatigue, thus causing SDB [35–37]. In addition, the immune response to antigens and other inflammatory stimuli in children with rhinitis causes lymphocyte hypertrophy. It stimulates tonsil and adenoid hyperplasia, which leads to obstruction of the nasopharyngeal and oropharyngeal passages and causes SDB [38, 39].

GER is a physiological process of retrograde entry of gastric contents into the esophagus due to relaxation of the lower esophageal sphincter. During wakefulness, it often occurs after meals and is rapidly cleared by swallowing, while during sleep, it occurs upon awakening. When it occurs in children, the throat is closed to prevent asphyxia, leading to apnea, thereby increasing the risk of SDB [40]. Co-existing GER increases the prevalence of SDB, and its treatment can improve SDB symptoms [41–43]. The adenoid is a lymphatic tissue located at the junction of the parietal and posterior walls of the nasopharynx and is part of the inner pharyngeal lymphatic ring. Adenoid hypertrophy is a leading risk factor for SDB [44, 45]. The adenoid size is associated with the severity of SDB [46–48]. Our findings also suggest that adenoid hypertrophyis a risk factor for SDB.

The tonsil is the first line of defense of human immune organs and is susceptible to bacterial and viral attacks. Chronic tonsillitis and SDB can coexist [49]. Sarmiento et al. [50] confirmed that B-cell hyperplasia and hypertrophy caused by OSAS result from chronic tonsillitis. Our findings also supported that chronic tonsillitis is a risk factor for SDB. In addition, LingShen et al. found that OSAS in children was closely associated with upper respiratory tract infections [47], consistent with our results, probably because they can increase inflammatory factors and promote tonsillar hypertrophy, thus causing or aggravating SDB.

In the present study, the family history of snoring was a risk factor for SDB, consistent with a previous report [37]. This finding suggested that family characteristics, such as body fat distribution and craniofacial features, may be involved in the pathogenesis of SDB [51].

Epidemiological studies have shown that asthma is increasing in prevalence yearly and has become a severe public health concern. In Chinese children, asthma is not well-controlled overall. In this study, 19.6% of children had poorly controlled asthma, lower than the rate reported from Turkey (39.5%) [27] but higher than that reported from South Korea (9.1%) [10]. The univariate and multivariate analyses of children with poorly controlled asthma showed that SDB and irregular medication use were independent risk factors for poor asthma control.

This study found that SDB was associated with not only poor asthma control, but also asthma severity, consistent with previous studies [9, 11, 12, 27, 52]. This may be because SDB can change the oropharyngeal reflex and increase intrathoracic negative pressure and airway vagus nerve tension, resulting in bronchial contraction, thus aggravating asthma. Local and systemic inflammatory responses are also involved [36]. Children with SDB repeatedly snore, leading to intermittent vibrations of the upper airways and soft tissue damage, resulting in local inflammation and edema. According to the united airway disease hypothesis, repeated snoring can theoretically lead to lower airway inflammation and worsening of asthma [53].

In the present study, 26.9% of children did not adhere to the prescribed medication for asthma control. The risk of poor asthma control was 1.571 times higher in children who did not take the prescribed medication regularly than those who did, suggesting that poor medication compliance is a critical factor affecting asthma control, consistent with previous reports [54–56]. Therefore, in clinical practice, individualized interventions should be undertaken to improve treatment compliance by changing the cognition of children and parents.

The strengths of this study include the reports of the prevalence and risk factors of SDB in children with asthma. Further, this was the first study on SDB and asthma control in China. However, this study has some limitations. First, this was a single-center, small-scale, cross-sectional observational study, which might be associated with selection bias and recall bias. In the future, prospective and cohort studies should be performed to validate that treating SDB can improve asthma control. Second, the risk of SDB was determined by PSQ-SRBD instead of objective tools (e.g., polysomnography), thus we could not make a definite diagnosis of SDB and could not determine the SDB subtypes, such as OSA and central sleep apnea (CSA).

## Conclusions

In summary, SDB is closely related to childhood asthma, and the combination of SDB may affect the severity and clinical control of asthma. We found that allergic rhinitis, chronic tonsillitis, gastroesophageal reflux, adenoid hypertrophy, recurrent respiratory tract infection, family snoring history are independent risk factors for SDB in children with asthma. Therefore, in clinical practice, for asthmatic children combined with these risk factors, it is recommended to screen SDB routinely in order to provide better treatment strategies for asthma control.

## Data Availability

The data that support the findings of this study are not openly available because they are human data and are available from the corresponding author upon reasonable request.
